# Biopsy variability of lymphocytic infiltration in breast cancer subtypes and the ImmunoSkew score

**DOI:** 10.1038/srep36231

**Published:** 2016-11-04

**Authors:** Adnan Mujahid Khan, Yinyin Yuan

**Affiliations:** 1 Centre for Evolution and Cancer, The Institute of Cancer Research, London, UK; 2 Division of Molecular Pathology, The Institute of Cancer Research, London, UK; 3 Centre for Molecular Pathology, Royal Marsden Hospital, London, UK

## Abstract

The number of tumour biopsies required for a good representation of tumours has been controversial. An important factor to consider is intra-tumour heterogeneity, which can vary among cancer types and subtypes. Immune cells in particular often display complex infiltrative patterns, however, there is a lack of quantitative understanding of the spatial heterogeneity of immune cells and how this fundamental biological nature of human tumours influences biopsy variability and treatment resistance. We systematically investigate biopsy variability for the lymphocytic infiltrate in 998 breast tumours using a novel virtual biopsy method. Across all breast cancers, we observe a nonlinear increase in concordance between the biopsy and whole-tumour score of lymphocytic infiltrate with increasing number of biopsies, yet little improvement is gained with more than four biopsies. Interestingly, biopsy variability of lymphocytic infiltrate differs considerably among breast cancer subtypes, with the human epidermal growth factor receptor 2-positive (HER2+) subtype having the highest variability. We subsequently identify a quantitative measure of spatial variability that predicts disease-specific survival in HER2+ subtype independent of standard clinical variables (node status, tumour size and grade). Our study demonstrates how systematic methods provide new insights that can influence future study design based on a quantitative knowledge of tumour heterogeneity.

Increasing evidence supports the use of lymphocytic infiltrate to predict prognosis and response to treatment in different cancer types^1^,^2^,^3^,^4^,^5^,^6^. In breast cancer, large-scale studies have shown consistent results, that high lymphocytic infiltrate estimated in histology tissue biopsies can predict overall survival^7^,^8^, response to adjuvant^7^,^9^,^10^ and neoadjuvant therapies^2^,^3^,^11^ in specific subtypes. As a result, international guidelines are being established to score lymphocytic infiltrate using Haematoxylin & Eosin (H&E)-stained tumour biopsies^5^. However, the amount of tissue needed to obtain target prognostic or predictive power remains unclear.

Tissue microarray (TMA) has played an important role in morphological and molecular analysis of tumour lymphocytic infiltrate^3^,^12^,^13^,^14^,^15^. Lymphocytes distribute differently among tumours and, within a tumour, depending on the spatial location, different numbers and types of immune cells may be presented^6^,^15^. Therefore, spatial heterogeneity of lymphocytic infiltrate is a critical factor that could confound studies that base their findings on TMAs. Thus far, comparisons between TMAs and whole-tumour sections have been performed with respects to quantification of markers such as Ki67^16^,^17^,^18^. However, thus far little is known about the level of variability in lymphocytic infiltration presented in biopsies in tumours in general. Even less is known about how such variability changes among breast cancer subtypes known to be very different diseases^19^,^20^. Moreover, traditional approaches that compare TMAs with whole sections generated from different part of tumour could be confounded by spatial differences within the tumour.

We therefore proposed a new approach that is fully automated and objective to evaluate biopsy variability of lymphocytic infiltrate across 998 breast cancers. Our aims were: (1) to evaluate the amount of TMA cores required for a reliable estimate of lymphocytic infiltrate; (2) to quantify variability in lymphocytic infiltrate scoring using TMA cores across different breast cancer subtypes; (3) to define the clinical significance of such variability in lymphocytic infiltration.

## Results

### Spatial heterogeneity of lymphocytic infiltrate captured by biopsies varies among patients

**Figure 1:**
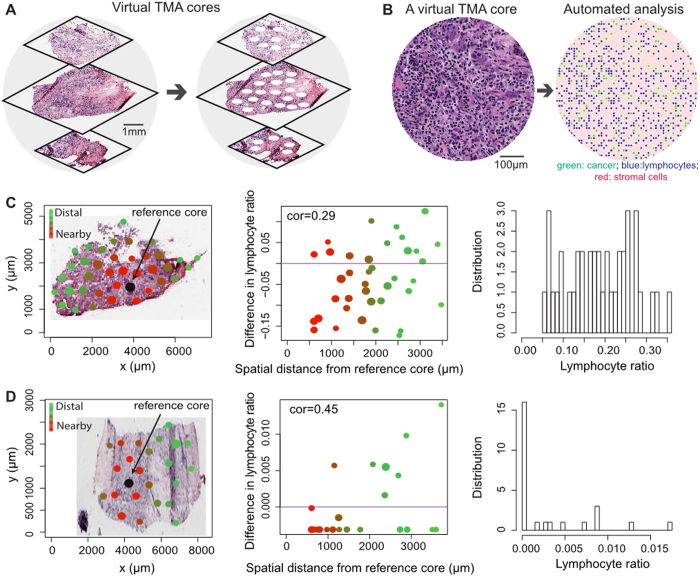
Spatial heterogeneity of lymphocytic infiltrate captured by biopsies varies among patients. (**A**) Illustration of the virtual biopsy pipeline. Three whole-tumour H&E stained sections to represent a primary breast tumour in the METABRIC study. Virtual, non-overlapping TMA cores were taken from this tumour. (**B**) An example of a virtual TMA core image, and identified cells after automated image analysis (green dots: cancer cells; red dots: stromal cells; blue dots: lymphocytes). (**C**,**D**) Illustration of tumour samples with low and high spatial correlation in lymphocytic infiltrate. For both samples, the left panel demonstrates the TMA cores sampled from a tissue section. Black coloured circle represents the reference core to compare against, the variation of colours between red and green shows the increase in distance from reference core (red means TMAs are closer to reference core while green means the core is relatively far away from reference core). Size of each circle scales with the number of cells in each TMA core. The middle panel plots the distance between each core from reference core against the difference in lymphocyte ratio between each core and the reference core. The right panel shows the marginal distribution of lymphocyte ratio in the cores.

To evaluate if TMA cores can provide a reliable estimate of the degree of lymphocytic infiltration in the whole tumour, we developed a virtual biopsy approach and applied it to 998 primary, untreated breast tumours (Fig. 1A, Table S1, Methods). On average three representative whole-tumour H&E sections were available for each tumour (Methods). Using this approach, non-overlapping tumour regions of size of a TMA core (0.6 mm in diameter) were randomly sampled in digital images of these sections. Previously, both intra-tumoural and stromal lymphocytes were reported to have prognostic or predictive value in breast cancer^3^,^7^,^21^, therefore we did not discriminate between stromal and tumour core regions for this discovery study. Virtual TMAs and whole-tumour section images were analysed by automated image analysis to identify cancer cells, lymphocytes and stromal cells that include fibroblasts and endothelial cells (Fig. 1B, Methods). At initial examination, we found that, within the same tumour, virtual cores can differ significantly in immune composition measured by the ratio of lymphocytes to all cells (lymphocyte ratio, Methods). Moreover, the differences among cores cannot always be explained by their spatial distances. For example, when all the TMA cores were compared with a reference core in the same tumour, which is a randomly selected core to compare against, a weak correlation between their spatial distances and differences in lymphocyte ratio was found in one case (Pearson correlation or cor = 0.29), where nearby cores can have very different levels of lymphocyte ratio (Fig. 1C). In another case, a higher correlation was found (cor = 0.45, Fig. 1D). These exhibits suggest that spatial heterogeneity of lymphocytic infiltrate varies among patients, and motivated us to further quantify the level of heterogeneity and how this differs among patients.

### Subtype-dependent concordance in lymphocytic estimate based on biopsies

**Figure 2:**
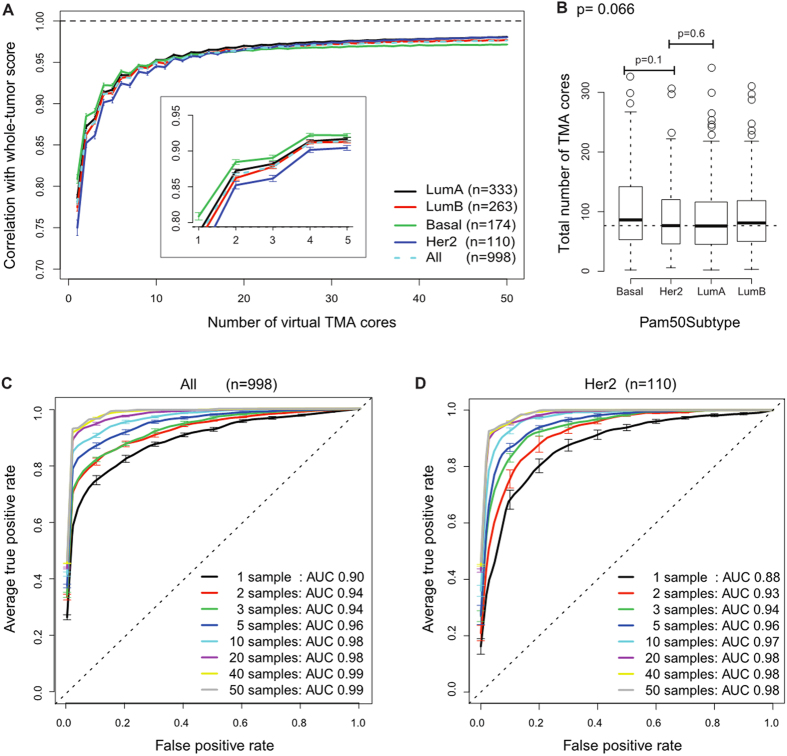
The number of TMA biopsies needed for estimating lymphocytic infiltrate varies among breast cancer subtypes. (**A**) Correlation between core-based and tumour-based scores of lymphocyte ratio over increasing number of cores for each subtype individually and combined. A magnified view of the same plot (n = 2–5) is inset for better visualisation. Difference in subtype specific correlation between HER2 and other subtypes is statistically significant (*n* = 4: HER2 versus Luminal A *p* = 0.010; HER2 versus Luminal B *p* = 0.010; HER2 versus Basal *p* = 0.011; one-sided paired t-test). (**B**) Boxplot to show the total number of virtual TMA cores for tumours in each subtype. The horizontal dash line marked the median value for HER2 subtype. (**C**) AUC curves for taking increasing amount of TMA cores for all unselected tumours. (**D**) AUC curves for taking increasing amount of TMA cores for HER2 tumours.

To quantify the heterogeneity of lymphocytic infiltrate in biopsies, we computed lymphocyte ratio score, defined as the ratio of lymphocytes to all cells in each virtual TMA core (core-based lymphocyte ratio) or whole-tumour section (tumour-based lymphocyte ratio, Methods). As expected, we observed an increased correlation between core-based and tumour-based scores of lymphocyte ratio with more cores (cor = 0.78 for *n* = 1, cor = 0.87 for *n* = 2, cor = 0.88 for *n* = 3), and this correlation reached a plateau at 0.96–0.98 when number of cores *n* exceeds 15 (Fig. 2A, Table S2, Methods).

We then asked if this correlation differs among breast cancer subtypes. We observed significantly lower correlations between core-based and tumour-based scores for the HER2 subtype compared with other PAM50 intrinsic subtypes with less than 10 cores (Fig. 2A; *n* = 4: HER2 versus Luminal A *p* = 0.010; HER2 versus Luminal B *p* = 0.010; HER2 versus Basal *p* = 0.011; one-sided paired t-test). This suggests that more cores are required to estimate lymphocytic infiltrate in HER2, possibly due to a higher level of intra-tumour heterogeneity in lymphocytic infiltrate (Fig. 2A). Basal subtype, on the other hand, had the highest correlation between core-based and tumour-based scores. To exclude the possibility that tumour size cofounded our analysis, since the bigger the tumour the more the TMA cores to sample from, we examined the numbers of TMA cores for each subtype. The numbers of cores in HER2 or Basal tumours were not higher or lower compared with other subtypes (*p* > 0.1, Kruskal-Wallis test), supporting that the low correlation between core-based and tumour-based scores we observed in HER2 tumours was not due to tumour size but likely reflected a higher level of intra-tumour heterogeneity (Fig. 2B).

### Marginal improvement of tumour classification accuracy with ≥4 cores

Next, we tested the accuracy in classifying tumours into low- or high- lymphocytic infiltrate based on TMA scores (Methods). Again classification based on tumour-based scores was used as gold standard. When all subtypes were combined, we observed an improvement from *n* = 1 core to *n* = 2 (area under the receiver operating characteristic curve (AUC) 0.90 to 0.94) and *n* = 3 to *n* = 4 (AUC 0.94 to 0.96), but no difference between *n* = 2 and *n* = 3 (AUC 0.94 and 0.94 respectively; Fig. 2C). When *n* ≥ 4, only marginal improvement in classification accuracy can be observed considering the amount of cores being added (AUC = 0.97 for *n* = 6–9). In the HER2 subtype, lower accuracy of core-based classification was found in general compared with the scores obtained from other subtypes combined (*n* = 1: HER2 AUC = 0.88 versus other subtypes AUC = 0.90, *n* = 4: HER2 AUC = 0.95 versus other subtypes AUC = 0.96, Fig. 2D, Table S2), consistent with our previous observation of higher spatial heterogeneity in this subtype.

### Prognostic value of core-based lymphocyte ratio

**Figure 3:**
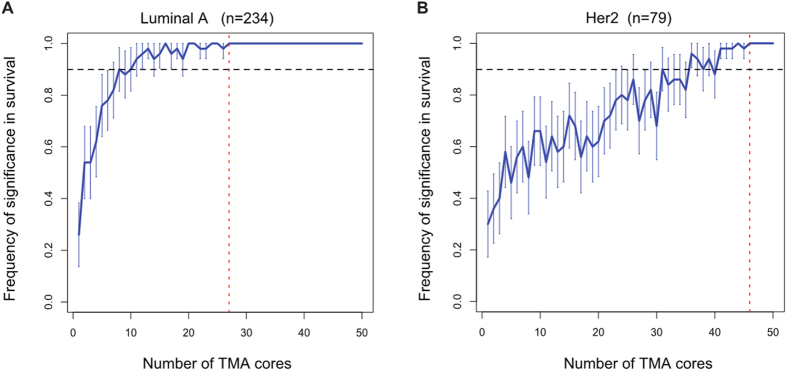
Increased stability in prognostic value of lymphocytic infiltrate score with more TMA cores for (**A**) Luminal A tumours and (**B**) HER2 tumours. Frequency or percentage of times where core-based score was found to be significantly associated with disease-specific survival in 100 random sampling experiments was calculated for an increasing number of cores. Red vertical dash line marks the core number where frequency first reached and stayed at 100%, and black horizontal line marks 90% frequency.

High tumour-based lymphocyte ratio scores was associated with favourable disease-specific survival in Luminal A and HER2 subtypes but not Basal and Luminal B (Luminal A: *p* = 0.0087, hazard ratio (HR) = 0.32, 95% confidence interval (CI) = [0.14, 0.75]; HER2: *p* = 0.047, HR = 0.51, CI = [0.27, 0.99]; combined cohorts, Fig. S1). We evaluated the prognostic value of core-based scores for these subtypes by varying the number of biopsies used with 50 repeated sampling (Methods). This left us with 234 samples of Luminal A subtype and 79 samples of HER2 subtype. For both subtypes, frequency of times when core-based scores were found to be prognostic increased with the number of cores used (Fig. 3A–B). For the frequency to be >90%, more than 10 cores were required for Luminal A but more than 35 cores were required for HER2, consistent with our previous observation that HER2 may be more heterogeneous.

### Biopsy variability of lymphocytic infiltrate is prognostic in HER2 subtype

**Figure 4:**
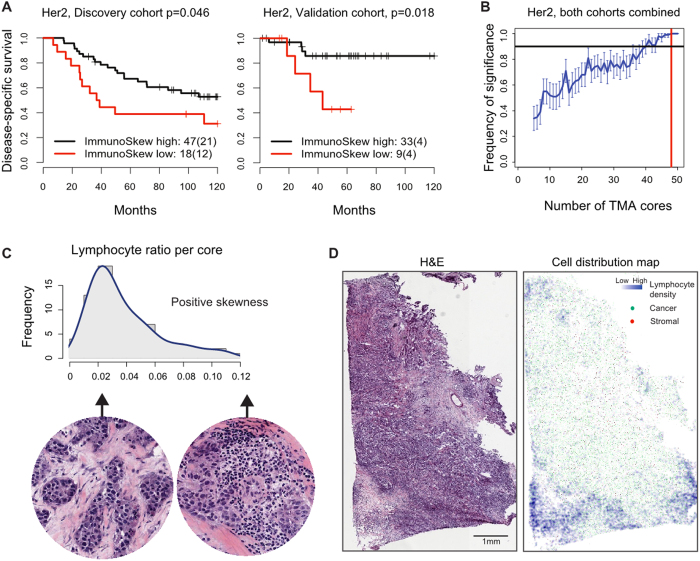
Reproducibility and stability of the ImmunoSkew score in HER2 breast cancer subtype. (**A**) Kaplan-Meier curves to illustrate disease-specific survival of patients with high versus low ImmunoSkew score in HER2 subtype for the Discovery cohort and Validation cohort. Shown in legend is the number of patients (the number of disease-specific events) per group. (**B**) Increased stability in the prognostic significance of ImmunoSkew scores with progressively more TMA cores. Red vertical dash line marks 48 TMA cores and black horizontal dash line marks 90% frequency. (**C**) An example tumour with high ImmunoSkew score as a result of high skewness in the distribution of core-based lymphocyte ratio. (**D**) An H&E image and corresponding cell distribution map from a tumour with high ImmunoSkew score.

Since variability in core biopsies represents intra-tumour heterogeneity, we tested its prognostic value against 10-year disease-specific survival (Methods). We quantitatively defined variability in core-based lymphocyte ratio by examining the score distribution of 50 randomly sampled cores from each tumour. We used two different variability measures: (1) standard deviation to define deviation from mean, and (2) skewness to measure the degree of asymmetry in a distribution. First, we found that standard deviation was not associated with survival in any subtype. In contrast, high skewness was associated with poor prognosis in the HER2 subtype (Discovery: *p* = 0.045, HR = 2.03, CI = [1.0073, 4.14]; Validation: *p* = 0.018, HR = 4.57, CI = [1.14, 18.34]; Fig. 4A), but not in other subtypes. We then varied the number of cores used for calculating this skewness score (*n* = 5–50), and found that the frequency of skewness being prognostic was 100% upon resampling when more than 48 cores were used (Fig. 4B). Higher degree of skewness was found in distributions that had a longer right tail than the left tail. In our study this translates into the situation where a small number of tumour regions have high percentage of lymphocytes while most regions have low percentage of lymphocytes, as illustrated in Fig. 4C–D. The association of high skewness with poor prognosis in HER2 may reflect ineffective immune response manifested as partial lymphocytic infiltrate into a tumour. We henceforth referred to this prognostic score as the ImmunoSkew score.

### ImmunoSkew is an unfavourable prognostic factor independent of clinical variables and treatment regimen in HER2 subtype

**Figure 5:**
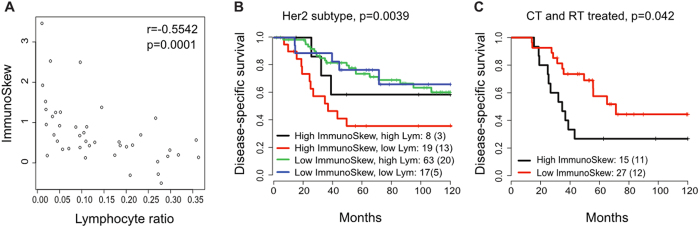
ImmunoSkew, lymphocyte ratio and treatment outcome. (**A**) A negative correlation between ImmunoSkew and lymphocyte ratio. (**B**) ImmunoSkew further stratifies patients grouped by lymphocyte ratio, particularly in the low lymphocyte ratio group. (**C**) In HER2 patients treated by both chemotherapy (CT) and radiotherapy (RT), ImmunoSkew identified a subgroup with very poor disease-specific survival.

The ImmunoSkew score was not significantly associated with clinicopathologic variables including lymph node status, tumour size, oestrogen receptor (ER) and HER2 status, molecular subtypes including PAM50 and IntClust integrative subtypes (*p* > 0.05, Table S1). It was, however, negatively correlated with tumour-based lymphocyte ratio (Discovery: r = −0.50, *p* = 1.1 × 10^−5^; Validation: r = −0.55, *p* = 1.4 × 10^−4^; Fig. 5A). This is expected as ImmunoSkew score measures spatial heterogeneity of immune cells, and therefore, a sample with prominent immune infiltrate will receive a low ImmunoSkew score. Nevertheless tumour-based lymphocytic ratio was prognostic in HER2 breast cancer, and the addition of ImmunoSkew to the low lymphocytic infiltrate group further stratified patients with statistical significance (blue vs red groups: *p* = 0.0039, Fig. 5B). Due to small number of samples in one of the cohorts (*n* = 42), multivariate analysis did not converge when analysis was performed on individual cohorts and further division of HER2+ samples into HER2+/ER+ versus HER2+/ER− subtypes was not possible. Therefore, multivariate analysis was performed using all HER2 samples (*n* = 110) to include node status, tumour size and grade, demonstrating that the ImmunoSkew score is independently prognostic in HER2 breast cancer (*p* = 0.02, HR = 1.51, CI = [1.07, 2.13]; Table 1).

Moreover, the addition of the ImmunoSkew score to HER2 patient groups defined by treatment regimen resulted in new sub-groups with substantially different disease-specific survival. When ImmunoSkew was used to stratify the patients treated by both chemotherapy and radiotherapy, which is the largest group (42 out of 110), we found that patients with high ImmunoSkew score had significantly worst prognosis compared with patients with low ImmunoSkew score, despite having aggressive treatments (*p* = 0.042, Fig. 5C). These analyses indicated the potential usage of ImmunoSkew score in addition to existing clinical variables for HER2 breast cancer.

## Discussion

The use of TMA has contributed to many key discoveries in tumour immune response over the last decade^3^,^12^,^13^,^14^,^15^. However, variability in the presence of immune cells in TMAs due to sampling of a small region could have profound implication on study outcome. In this study, we developed a virtual biopsy approach and designed the first large-scale study, to our knowledge, to systematically evaluate the degree of variability in lymphocyte ratio captured by TMA cores in breast cancers. We constructed virtual TMA cores (core size = 0.6 mm) from multiple whole-tumour sections that represent the same tumour, estimated lymphocytic infiltrate as the ratio of lymphocytes to all cells using automated histology image analysis and evaluated the performance of core-based lymphocyte ratio scores as a function of core numbers. In unselected breast cancers, whilst we observed a fair degree of concordance between core-based and tumour-based scores with only 1 biopsy (cor = 0.78, AUC = 0.90), the concordance soon increased with 2 biopsies (cor = 0.87, AUC = 0.94) and continued to rise until plateau soon after 4 cores (cor = 0.91, AUC = 0.94 for *n* = 4, AUC = 0.97 for *n* = 6–9). Therefore, we expect a substantial improvement in the estimation of immune infiltrate with the use of 2 TMA cores compared with 1 core; the use of 4 cores represents a good trade-off between performance and the amount of tissue required; however, only marginal improvement in performance can be achieved with much more tissue.

Interestingly, significant variation in concordance between core-based and tumour-based lymphocyte ratio was identified according to breast cancer subtypes. We found lower correlation between core-based and tumour-based scores in HER2 compared with other PAM50 intrinsic subtypes (Fig. 2A,C,D). HER2 tumours were not larger or smaller than other subtypes (*p* > 0.05, Fig. 2B), suggesting that the lower correlation cannot be attributed to tumour size but is likely due to a higher degree of spatial heterogeneity of lymphocytic infiltrate in this subtype. Next, we investigated the prognostic significance of lymphocyte ratio in breast cancer subtypes and found positive association between high lymphocyte ratio and favourable 10-year disease-specific survival in both Luminal A (*p* = 0.0087, HR = 0.32, 95% CI = [0.14, 0.75]) and HER2 subtypes (*p* = 0.047, HR = 0.51, 95% CI = [0.027, 0.99]). Consistent with our findings above, we observed that more cores are needed for the HER2 subtype to gain consistent prognostic value compared with the Luminal A subtype (38 versus 25 cores). Our observation emphasises the need to control how sampling is performed especially for HER2 subtype. This appears to be in contrast to recent studies that estimate TILs from a single TMA core^22^,^23^. However, the proportion of HER2 tumours in these studies was significantly lower, which may explain the good prognostic value of TILs observed in these studies. Our results therefore suggest that more cores should be considered for the HER2 subtype during study design, compared with other subtypes. For example, to achieve the same performance with 4 cores in breast tumours from other subtypes (Basal, Luminal A and Luminal B), 6 cores would be needed for the HER2 subtype (HER2 *n* = 6: cor = 0.92, AUC = 0.96; other subtypes *n* = 4: cor = 0.92, AUC = 0.96; Table S2).

Our study to examine biopsy variability led to the discovery of a quantitative measure of spatial heterogeneity in lymphocytic infiltrate, namely the ImmunoSkew score. High ImmunoSkew was found to be associated with poor prognosis in the HER2 subtype (Discovery: *p* = 0.045, HR = 2.03, CI = [1.01, 4.14]; Validation: *p* = 0.018, HR = 4.57, CI = [1.14, 18.34]). Multivariate analysis also demonstrated the value of the ImmunoSkew as a prognostic factor independent of other clinical parameters (node, size, grade) in HER2 subtype (*p* = 0.02, HR = 1.51, CI = [1.07, 2.13]). Combining ImmunoSkew with treatment regimen including chemotherapy and radiotherapy, we found that HER2 patients with high ImmunoSkew had significantly worst prognosis compared with patients with low ImmunoSkew, irrespective of treatments (*p* = 0.048, HR = 0.44, CI = [0.19, 0.99]). High ImmunoSkew score reflects a strong regional segregation of immune cells, and its association with poor prognosis may indicate dysfunctional immune infiltrate. Given the increasing amount of evidence that immune infiltrated HER2+ tumours are more sensitive to chemotherapy and other therapies^24^, our data underscore the importance of considering the spatial variability of immune cells besides their abundance for developing new predictive biomarkers. On a broader scale, our finding is in line with previous observations that immune response as a cancer hallmark is often predictive of cancer prognosis^25^, not only in breast cancer^21^,^26^,^27^ but also in other cancer types^28^.

Since our samples were collected, the standard of care for HER2+ patients has changed with the introduction of HER2-targeted therapies. Therefore, the prognostic effect of ImmunoSkew remains to be verified under the current treatment routine. However, an active immune response is known to be critical for HER2-inibition treatment^7^,^24^,^29^,^30^. The need to understand the collective biological nature of the immune microenvironment in HER2 breast cancer, defined by spatial variability, type and abundance of immune cells, as well as their concomitant effect on current and future treatments including immunotherapy, will shape future research on this aggressive cancer type.

A limitation of our study is the use of 2D images to represent a 3D tumour structure. To mitigate this issue, tumour sections from top, middle and bottom part of a tumour were analysed to recapitulate a situation where TMA cores were sampled from the whole tumour. Our method for estimating lymphocytic infiltrate is different from the current recommendations by TILs working group^5^. For instance, as per recommendations, TILs should be reported in the stromal compartment, within the borders of the invasive tumour particularly excluding the regions around ductal carcinoma *in situ* and normal lobules. Such considerations were not taken into account in the current study since we have taken an unbiased approach of tissue sampling to gain an understanding of lymphocyte distribution within breast tumours. Other studies sampled TMAs from tumour periphery and tumour core^29^,^30^. The development of an automated system to identify these tumour regions, coupled with our virtual biopsy approach, will allow us to refine our findings with reproducibility and warrants further investigation.

Despite these limitations, our fully automated analysis of digital histological sections provides an objective approach towards the *de novo* assessment of spatial variability including but not limited to the estimation of lymphocytic infiltrate. Extensions to include various sizes of TMAs, other types of biopsies such as needle biopsies, will enhance our knowledge on the limitations of tumour biopsies. We expect studies like this will provide valuable information for future design of studies or clinical trials.

## Methods

### Clinical samples

The METABRIC dataset consists of a total of 1,980 primary treatment-näive breast tumours from five contributing hospitals, with patient consents and ethical approval by relevant review boards as reported in our previous publication^20^. Samples from two of the five hospitals were not found suitable for automated analysis, since there were severe artefacts mainly because of long-term storage of frozen tissues. Samples from the remaining three hospitals (998 samples, sample characteristics in Table S1) were considered for this study. Samples from the two hospitals were combined to form a Discovery cohort (Cohort 1 = 484 patients), while the samples from the third hospital were used as an independent Validation cohort (Cohort 2 = 514 patients). On average, three sections were obtained from top, middle and bottom of each tumour block, placed on the same glass slide, stained with H&E and scanned at ×20 magnification using ScanScope TX scanner (Aperio Technology Inc.), which converted a glass slide into a digital histological image. The tumour material between these three sections was mixed and used for molecular profiling. For all of the selected samples, PAM50 subtyping status was determined based on expression microarray data. Tumour cellularity was scored into three categories: low (<40% tumour DNA), moderate (≥40% and <70% tumour DNA) and high (≥70% tumour DNA). Similarly, lymphocytic infiltration was scored into three categories by expert pathologists in the METABRIC consortium^20^: absent, mild and severe: absent if there were no lymphocytes, mild if there was a light scattering of lymphocytes, and severe if there was a prominent lymphocytic infiltrate. Information on treatment regime (chemotherapy, radiotherapy and hormone therapy) was available for all the selected patients. Patients were not treated by HER2-targeted therapy.

### Automated image analysis

Cancer cells are generally large in size and demonstrate greater variability in appearance as compared to the immune and stromal cells; lymphocytes typically are small, round and homogeneously basophilic and other cells (e.g. stroma, fibroblasts and endothelial cells) are more elongated. Our image analysis pipeline^8^ exploits these morphological differences among cancer cells, lymphocytes and other cells to accurately identify these primitives in an H&E stained histological tissue section. Broadly, the image analysis pipeline consisted of four stages: (1) unsupervised segmentation of the tissue primitives (cells); (2) supervised classification of individual cells into cancer cells, lymphocytes, other cells, and artefacts; (3) kernel smoothing to correct local sporadic errors; and (4) a hierarchical multiresolution model fitting to identify cancer cell clusters to further improve classification accuracy.

Segmentation was performed by employing Otsu thresholding followed by distance transform based watershed segmentation to separate the touching objects. Next, an expert pathologist hand annotated a set of 871 primitives (approximately 200 primitives from each of the four classes). For each of these hand annotated primitives, a dictionary of morphological and textural features was computed with an aim to represent each primitive in a high-dimensional feature space. Finally, a multiclass support vector machine (SVM) classifier with Gaussian kernel was trained to model the morphological differences among different tissue primitives. Once trained, the model was used to perform classification of all the primitives identified during stage 1 of the image analysis pipeline.

The kernel smoothing was then performed for cells in spatial proximity to cancer cells to remove erroneous erratic classifications. Finally, a multiresolution hierarchical model was employed to provide further improvement in classification accuracy by identifying cancer cell clusters instead of individual cells as identified in the previous steps. The entire image processing pipeline was supplied as an R package CRImage.

To evaluate the accuracy of our image analysis pipeline, we performed three set of experiments: (1) 10-fold cross validation within the training set (90.1% accuracy)^8^; (2) 10,000 single cell annotation by an expert pathologist in random samples (cor = 0.98)^8^; and (3) correlation with pathological score of tumour cellularity and lymphocytic infiltration (Jonckheere-Terpstra (JT) trend test *p* < 0.0001). Automated score of lymphocytic infiltrate was defined as the percentage of lymphocytes in all cells by image analysis, and it was found to be highly correlated with pathological score (*p* < 0.0001, JT trend test). On average there were 56,815 (mean) cancer cells (± standard deviation 54,400), 9,730 lymphocytes (±13,570) and 11,766 other cells (±10,791) in each tumour.

### Virtual biopsy

We generated virtual TMA cores from the H&E stained whole-tumour sections using the following computational algorithm.

Let 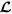 be the list containing the spatial locations of cells identified using image analysis, repeat the following three steps until 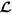 is not empty.

1. Get the first element of 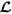, the spatial location of a cell, and consider it as a proposed centre of TMA core;
2. Count the number of cells within the circular region of radius 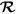 around the proposed centre;
3. If the total number of cells in the region is greater than 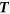, accept the proposed spatial location as the centre of TMA core and remove all the enclosed cell locations from 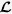; otherwise, drop the proposed TMA core and exclude the current spatial location from 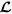.

Since a commonly used size of a TMA core is 0.6 mm, we fixed 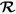 = 0.3 mm. Due to the lack of concrete clinical guideline for the parameter 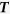, we empirically fixed its value to 100. Each core must have at least 100 cells, regardless of cell types, to be considered as a virtual TMA core. On average, we generated 88.53 (±58.01) virtual TMA cores per sample.

### Quantitative measures

We derived the following quantitative measures based on image analysis:

#### Tumour-based lymphocyte ratio

The ratio of the number of lymphocytes to the total number of cells present in all sections from a single tumour.

#### Core-based lymphocyte ratio

The ratio of the number of lymphocytes to the total number of cells present in a TMA core.

#### ImmunoSkew

The skewness of the distribution of core-based lymphocyte ratio in a tumour as a measure of spatial heterogeneity in lymphocytic infiltrate. Mathematically,


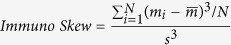


Where 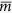 and 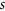 are the mean and standard deviation of core-based lymphocyte ratio, m_i_ is the i-th TMA core which varies between 1 to N, and N is the total number of virtual TMA cores.

#### Standard deviation of lymphocyte heterogeneity

The standard deviation of the core-based lymphocyte ratio as another measure of spatial heterogeneity in lymphocytic infiltrate.

### Comparison and survival analysis of biopsy-based scores

To compare core-based scores with tumour-based scores, we randomly sampled *n* cores and benchmarked the average lymphocyte ratio scores of these cores against the tumour-based scores. This was repeated 100 times for *n* = 1–50 for each tumour. For samples with less than 50 TMA cores, we performed sampling with replacement. To enable fair comparison across tumours during the survival analysis, *n* TMA cores were randomly sampled from each tumour and the average lymphocyte ratio score was evaluated in survival analysis. This was repeated 50 times for each *n*, *n *= 1–50. To ensure the results were comparable, we only used the subset of samples with at least 50 TMA cores.

### Other statistical methods

Associations between continuous variables were tested by Pearson’s correlation coefficient. Kruskal-Wallis test was used to assess the relationship between continuous and categorical variables. Two-sided paired t-test was used to assess the difference between the two matched continuous variables. To estimate the accuracy of core-based lymphocyte ratio in predicting immune abundance, we used AUC as a measure of accuracy, and random sampling to estimate 95% CIs. Univariate survival analysis was performed with disease specific 10-year survival data using the Kaplan-Meier method, where p-value was calculated using log-rank test. To dichotomise continuous measures including the ImmunoSkew score, we systematically evaluated every hundredth value of each of the above stated measure in the 20–80% range using univariate analysis. A value that best dichotomised survival in the Discovery cohort was selected as the cut-off value and was evaluated in the Validation cohort. Cox proportional hazard model was fitted to the survival data and HR as well as 95% CI was computed to determine the prognostic value. Multivariate analysis was performed using Cox proportional hazard model for predictors found to be statistically significant in univariate analysis. For all statistical tests, *p*-value < 0.05 was considered to be statistically significant.

## Conclusions

In summary, by employing digital image analysis and machine learning, we sought to determine the number of cores needed for an estimate of immune infiltrate in breast tumours. Virtual TMAs were constructed from whole-tumour histology sections and systematically compared. We revealed that HER2+ breast tumours have a higher degree of spatial heterogeneity of lymphocytic infiltrate and therefore require more TMA cores compared with the tumours of other subtypes. This biological nature of HER2 subtype further motivated the discovery of ImmunoSkew, a quantitative measure of intra-tumour heterogeneity of lymphocytic infiltrate that can potentially serve as a prognostic biomarker in HER2 breast cancer based on routinely generated histology samples.

## Additional Information

**How to cite this article**: Khan, A. M. and Yuan, Y. Biopsy variability of lymphocytic infiltration in breast cancer subtypes and the ImmunoSkew score. *Sci. Rep.* **6**, 36231; doi: 10.1038/srep36231 (2016).

**Publisher’s note:** Springer Nature remains neutral with regard to jurisdictional claims in published maps and institutional affiliations.

## Supplementary Material

Supplementary Information

## Figures and Tables

**Table 1:** Univariate and multivariate analysis of ImmunoSkew index using Cox proportional hazards model to predict disease-specific survival in HER2 breast cancer patients (*n* = 110).

	Univariate Analysis			Multivariate Analysis		
	*p*-value	HR	HR 95% CI	*p*-value	HR	HR 95% CI
Node Metastasis	0.00018*	4.77	2.10–10.82	0.0033*	3.52	1.52–8.18
Tumour Size	0.0031*	2.14	1.29–3.56	0.044*	1.71	1.01–2.91
Tumour Grade	0.73	1.12	0.58–2.16	0.93	0.97	0.49–1.92
**ImmunoSkew**	0.0031*	**2.58**	**1.37**–**4.85**	0.040*	**1.97**	**1.02**–**3.79**
Lymphocytic Infiltrate	0.050*	0.53	0.29–1.00	0.38	0.73	0.37–1.46
**ImmunoSkew**	—	—	—	0.022*	**2.26**	**1.12**–**4.55**
Treatment	2.36 × 10^−12^*	4.82	3.11–7.50	4.66 × 10^−12^*	4.74	3.05–7.36
**ImmunoSkew**	—	—	—	0.00027*	**1.74**	**1.29**–**2.36**

^*^Statistical significance.
