# Supplementary material for: Biopsy variability of lymphocytic infiltration in breast cancer subtypes and the ImmunoSkew score
Source: Sci Rep. 2016 Nov 4;6:36231. doi: 10.1038/srep36231 (PMC5095894; doi:10.1038/srep36231)
Supplement: Supplementary Information [file srep36231-s1.pdf]

Biopsy variability of lymphocytic infiltration in breast cancer subtypes and the ImmunoSkew score

Supplementary Material

Adnan Mujahid Khan[1, 2, 3], Yinyin Yuan[1, 2, 3*]

[1] Centre for Evolution and Cancer, The Institute of Cancer Research, London, UK

[2] Division of Molecular Pathology, The Institute of Cancer Research, London, UK

[3] Centre for Molecular Pathology, Royal Marsden Hospital, London, UK

*Corresponding author:

Yinyin Yuan, Ph.D., The Institute of Cancer Research, London, UK.

Tel: +44-20-89156632.

Email: Yinyin.Yuan@icr.ac.uk.

Supplementary Figure Legends

Figure S1. **Prognostic significance of tumour- and core-based lymphocyte ratio scores in Luminal A and HER2 breast cancers. A.** Kaplan-Meier curves to illustrate disease-specific survival of patients with high versus low tumour-based lymphocyte ratio in Luminal A subtype (HR=4.11, CI = [1.75, 9.63]). **B.** Kaplan-Meier curves to illustrate disease-specific survival of patients with high versus low core-based lymphocyte ratio in Luminal A subtype (HR=4.40, CI = [1.87, 10.37]). **C.** Kaplan-Meier curves to illustrate disease-specific survival of patients with high versus low tumour-based lymphocyte ratio in HER2 subtype (HR=3.00, CI = [1.60, 5.60]). **D.** Kaplan-Meier curves to illustrate disease-specific survival of patients with high versus low core-based lymphocyte ratio in HER2 subtype (HR=3.001, CI = [1.60, 5.60])

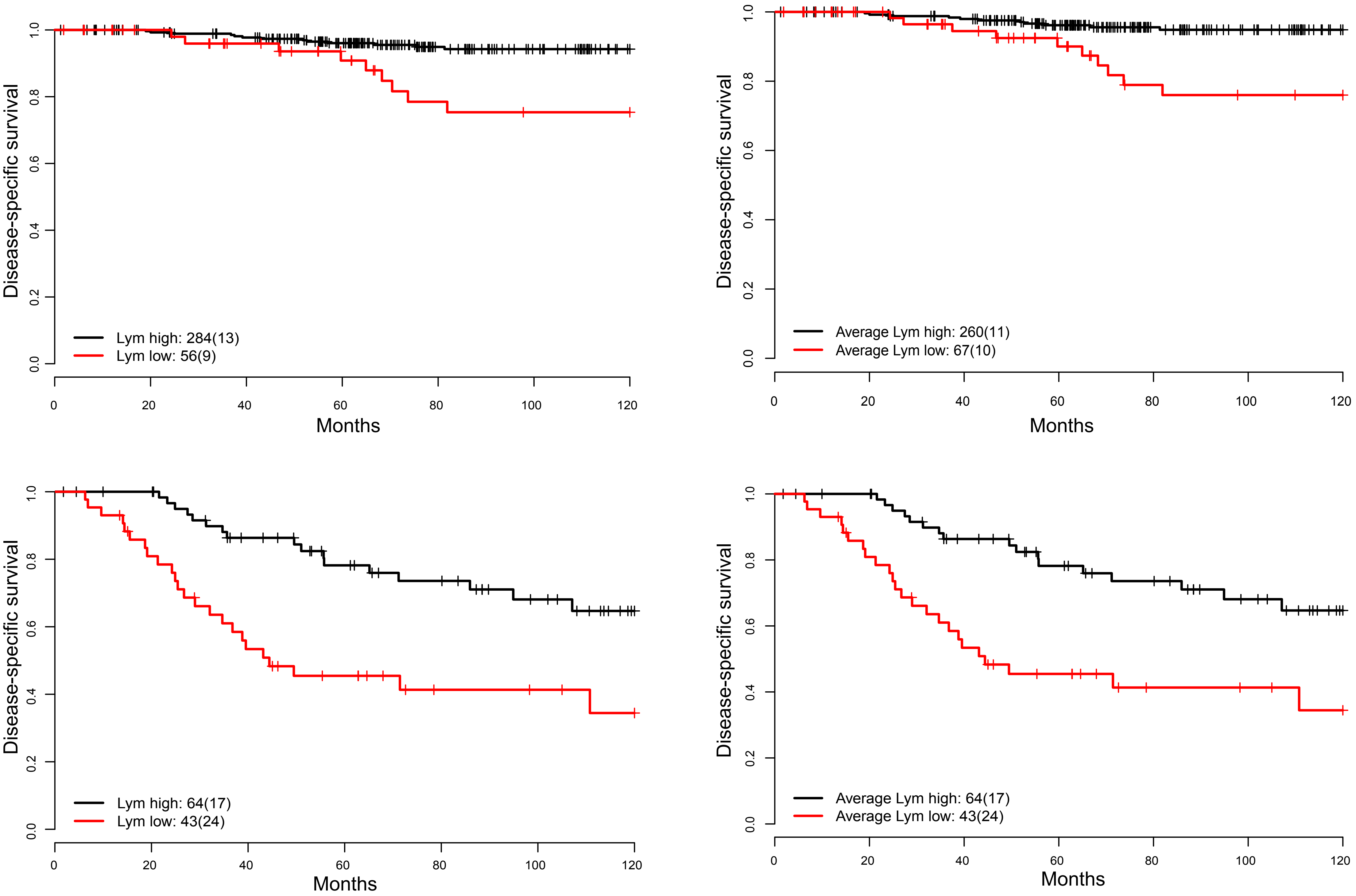

Disease-specific survival
1.0
0.8
0.6
0.4
0.2
0.0
0
20
40
60
80
100
120
Months
Lym high: 284(13)
Lym low: 56(9)
Average Lym high: 260(11)
Average Lym low: 67(10)
Lym high: 64(17)
Lym low: 43(24)
Average Lym high: 64(17)
Average Lym low: 43(24)

Supplementary Tables

Table S1: Distribution of the clinicopathological characteristics of all samples split into Discovery and Validation cohorts according to the ImmunoSkew index. Results are presented as number (%) or median (range). Death: breast cancer-specific death, Size: tumour size, Node: lymph-node status, Grade: tumour grade, ER: oestrogen receptor expression status defined by gene expression data, HER2: Human epidermal growth factor receptor-2 status defined by SNP6 copy number data, *TP53*: *TP53* mutation status, IA: immune abundance, Pam50: intrinsic subtypes. Samples with missing data were not shown. *p*-values are calculated by Kruskal-Wallis test or Fisher's exact test. *: statistical significance.

Factors		Discovery			Validation		
		Low ImmunoSkew	High ImmunoSkew	*p*	Low ImmunoSkew	High ImmunoSkew	
Number		322	162	-	375	139	-
Follow-up		118.3 (4.9-120)	111 (4.2-120)	-	62.5 (0.3-120)	45.5 (1.2-120)	-
Age		59.2 (27.6-86.1)	59.7 (21.9-83.4)	0.54	60.8 (30-96.3)	61 (26.4-90)	1
Survival	Alive	250 (77.6%)	107 (66%)	0.012*	334 (89.1%)	118 (84.9%)	0.17
	Death	66 (20.5%)	50 (30.9%)		40 (10.7%)	21 (15.1%)	
Size	<2cm	125 (38.8%)	62 (38.3%)	0.93	119 (31.7%)	42 (30.2%)	0.16
	>2cm, <5cm	180 (55.9%)	93 (57.4%)		226 (60.3%)	78 (56.1%)	
	>5cm	17 (5.3%)	7 (4.3%)		28 (7.5%)	18 (12.9%)	
Node	0	158 (49.1%)	77 (47.5%)	0.77	178 (47.5%)	58 (41.7%)	0.27
	1	164 (50.9%)	85 (52.5%)		193 (51.5%)	79 (56.8%)	
Grade	1	42 (13%)	13 (8%)	0.04*	38 (10.1%)	13 (9.4%)	0.85
	2	122 (37.9%)	54 (33.3%)		128 (34.1%)	52 (37.4%)	
	3	145 (45%)	94 (58%)		188 (50.1%)	68 (48.9%)	
ER	Neg	82 (25.5%)	51 (31.5%)	0.19	63 (16.8%)	20 (14.4%)	0.59
	Pos	234 (72.7%)	109 (67.3%)		295 (78.7%)	114 (82%)	
HER2	Neg	239 (74.2%)	120 (74.1%)	1	297 (79.2%)	111 (79.9%)	1
	Pos	83 (25.8%)	42 (25.9%)		77 (20.5%)	28 (20.1%)	
TP53	MUT	8 (2.5%)	10 (6.2%)	0.45	26 (6.9%)	10 (7.2%)	0.71
	WT	88 (27.3%)	68 (42%)		187 (49.9%)	86 (61.9%)	
IA	High	104 (32.3%)	131 (80.9%)	$7.2\text{x}10^{-25}$*	77 (20.5%)	105 (75.5%)	$2.6\text{x}10^{-30}$*
	Low	218 (67.7%)	31 (19.1%)		298 (79.5%)	34 (24.5%)	
Pam50	Basal	56 (17.4%)	41 (25.3%)	0.018*	57 (15.2%)	20 (14.4%)	0.87
	HER2	50 (15.5%)	18 (11.1%)		33 (8.8%)	9 (6.5%)	
	Luminal A	115 (35.7%)	40 (24.7%)		128 (34.1%)	50 (36%)	
	Luminal B	82 (25.5%)	50 (30.9%)		96 (25.6%)	35 (25.2%)	

Table S2: Concordance of core-based and tumour-based lymphocyte ratio scores as a function of core number used for: A. unselected samples versus HER2 subtype samples; **B.** samples from all subtypes combined (Basal, Luminal A, Luminal B and HER2) versus HER2 subtype samples; **C.** samples from all subtypes combined excluding HER2 subtype (Basal, Luminal A and Luminal B) versus HER2 subtype samples. Concordance was measured in terms of average correlation and average AUC scores. Average scores were computed over 100 repeats of sampling. Corresponding ranges are provided to demonstrate the variability within each concordance measure. AUC: Area under the receiver operating characteristic curve.

A. Concordance of core-based and tumour based lymphocyte ratio scores for unselected samples versus HER2 subtype samples.

No. of Biopsies	Correlation						AUC					
	All Subtypes (*n*=998)			HER2 (*n*=110)			All Subtypes (*n*=998)			HER2 (*n*=110)		
	Average	Range		Average	Range		Average	Range		Average	Range	
1	0.78	0.75	0.80	0.75	0.62	0.85	0.90	0.87	0.92	0.88	0.81	0.92
2	0.87	0.85	0.89	0.85	0.78	0.90	0.94	0.92	0.95	0.93	0.87	0.98
3	0.88	0.86	0.90	0.86	0.81	0.91	0.94	0.93	0.96	0.94	0.87	0.97
4	0.91	0.90	0.93	0.90	0.86	0.93	0.96	0.95	0.97	0.95	0.92	0.98
5	0.91	0.90	0.92	0.90	0.86	0.95	0.96	0.95	0.97	0.96	0.92	0.99
6	0.93	0.92	0.94	0.92	0.88	0.96	0.97	0.95	0.98	0.96	0.94	0.99
7	0.93	0.92	0.94	0.92	0.89	0.95	0.97	0.96	0.98	0.96	0.94	0.99
8	0.94	0.94	0.95	0.94	0.91	0.96	0.97	0.97	0.98	0.97	0.94	0.99
9	0.94	0.93	0.95	0.93	0.90	0.96	0.97	0.96	0.98	0.97	0.94	0.99
10	0.95	0.94	0.96	0.95	0.92	0.97	0.98	0.97	0.98	0.97	0.95	0.99
11	0.95	0.94	0.96	0.95	0.91	0.96	0.98	0.97	0.98	0.97	0.95	0.99
12	0.96	0.95	0.96	0.95	0.92	0.97	0.98	0.97	0.99	0.97	0.95	0.99
13	0.96	0.95	0.96	0.95	0.92	0.97	0.98	0.97	0.98	0.97	0.94	1.00
14	0.96	0.95	0.96	0.96	0.93	0.97	0.98	0.97	0.99	0.98	0.96	1.00
15	0.96	0.95	0.97	0.96	0.93	0.98	0.98	0.97	0.99	0.98	0.95	0.99
20	0.97	0.96	0.97	0.97	0.95	0.98	0.98	0.98	0.99	0.98	0.96	0.99
40	0.98	0.97	0.98	0.98	0.97	0.98	0.99	0.98	0.99	0.98	0.98	0.99
50	0.98	0.98	0.98	0.98	0.98	0.98	0.99	0.99	0.99	0.98	0.98	0.98

B. Concordance of core-based and tumour based lymphocyte ratio scores for all subtypes (Basal, Luminal A, Luminal B, HER2) versus HER2 subtype samples.

No. of Biopsies	Correlation						AUC					
	All Subtypes (*n*=770)			HER2 (*n*=110)			All Subtypes (*n*=770)			HER2 (*n*=110)		
	Average	Range		Average	Range		Average	Range		Average	Range	
1	0.78	0.75	0.81	0.75	0.62	0.85	0.90	0.87	0.92	0.88	0.81	0.92
2	0.87	0.85	0.89	0.85	0.78	0.90	0.94	0.92	0.96	0.93	0.87	0.98
3	0.88	0.86	0.90	0.86	0.81	0.91	0.94	0.92	0.96	0.94	0.87	0.97
4	0.91	0.90	0.93	0.90	0.86	0.93	0.96	0.95	0.97	0.95	0.92	0.98
5	0.92	0.90	0.93	0.90	0.86	0.95	0.96	0.94	0.97	0.96	0.92	0.99
6	0.93	0.92	0.94	0.92	0.88	0.96	0.97	0.95	0.98	0.96	0.94	0.99
7	0.93	0.92	0.94	0.92	0.89	0.95	0.97	0.96	0.98	0.96	0.94	0.99
8	0.94	0.93	0.95	0.94	0.91	0.96	0.97	0.96	0.98	0.97	0.94	0.99
9	0.94	0.93	0.95	0.93	0.90	0.96	0.97	0.96	0.98	0.97	0.94	0.99
10	0.95	0.94	0.96	0.95	0.92	0.97	0.98	0.97	0.98	0.97	0.95	0.99
11	0.95	0.94	0.96	0.95	0.91	0.96	0.98	0.97	0.98	0.97	0.95	0.99
12	0.96	0.95	0.96	0.95	0.92	0.97	0.98	0.97	0.99	0.97	0.95	0.99
13	0.96	0.95	0.96	0.95	0.92	0.97	0.98	0.97	0.98	0.97	0.94	1.00
14	0.96	0.95	0.97	0.96	0.93	0.97	0.98	0.97	0.99	0.98	0.96	1.00
15	0.96	0.95	0.97	0.96	0.93	0.98	0.98	0.97	0.98	0.98	0.95	0.99
20	0.97	0.96	0.97	0.97	0.95	0.98	0.98	0.98	0.99	0.98	0.96	0.99
40	0.98	0.97	0.98	0.98	0.97	0.98	0.99	0.98	0.99	0.98	0.98	0.99
50	0.98	0.98	0.98	0.98	0.98	0.98	0.99	0.99	0.99	0.98	0.98	0.98

C. Concordance of core-based and tumour based lymphocyte ratio scores for all subtypes excluding HER2 subtype samples (Basal, Luminal A, Luminal B) versus HER2 subtype samples.

No. of Biopsies	Correlation						AUC					
	All Subtypes (*n*=770)			HER2 (*n*=110)			All Subtypes (*n*=770)			HER2 (*n*=110)		
	Average	Range		Average	Range		Average	Range		Average	Range	
1	0.79	0.76	0.82	0.75	0.62	0.85	0.90	0.87	0.92	0.88	0.81	0.92
2	0.87	0.85	0.89	0.85	0.78	0.90	0.94	0.92	0.96	0.93	0.87	0.98
3	0.88	0.86	0.90	0.86	0.81	0.91	0.94	0.92	0.96	0.94	0.87	0.97
4	0.92	0.90	0.93	0.90	0.86	0.93	0.96	0.95	0.97	0.95	0.92	0.98
5	0.92	0.90	0.93	0.90	0.86	0.95	0.96	0.94	0.97	0.96	0.92	0.99
6	0.93	0.92	0.95	0.92	0.88	0.96	0.97	0.96	0.98	0.96	0.94	0.99
7	0.93	0.92	0.94	0.92	0.89	0.95	0.97	0.96	0.98	0.96	0.94	0.99
8	0.94	0.94	0.95	0.94	0.91	0.96	0.97	0.96	0.98	0.97	0.94	0.99
9	0.94	0.93	0.95	0.93	0.90	0.96	0.97	0.96	0.98	0.97	0.94	0.99
10	0.95	0.94	0.96	0.95	0.92	0.97	0.98	0.97	0.98	0.97	0.95	0.99
11	0.95	0.94	0.96	0.95	0.91	0.96	0.98	0.97	0.98	0.97	0.95	0.99
12	0.96	0.95	0.97	0.95	0.92	0.97	0.98	0.97	0.99	0.97	0.95	0.99
13	0.96	0.95	0.97	0.95	0.92	0.97	0.98	0.97	0.98	0.97	0.94	1.00
14	0.96	0.95	0.97	0.96	0.93	0.97	0.98	0.97	0.99	0.98	0.96	1.00
15	0.96	0.95	0.97	0.96	0.93	0.98	0.98	0.97	0.99	0.98	0.95	0.99
20	0.97	0.96	0.97	0.97	0.95	0.98	0.98	0.98	0.99	0.98	0.96	0.99
40	0.98	0.97	0.98	0.98	0.97	0.98	0.99	0.98	0.99	0.98	0.98	0.99
50	0.98	0.98	0.98	0.98	0.98	0.98	0.99	0.99	0.99	0.98	0.98	0.98
